# Some Interesting Points in Oral Surgery

**Published:** 1883-11

**Authors:** George L. Parmele


ARTICLE IV.

### SOME POINTS IN ORAL SURGERY OF INTEREST TO THE GENERAL PRACTITIONER.

BY GEORGE L. PARMELE, M. D., D. D. S.,

(Presented to the Ninety-Second Annual Meeting of the Connecticut Medical Society, May 23, 1883.)

I should much rather be a silent listener to words of wisdom and instruction from those older in membership in this Society and profession than myself, and consequently better qualified to be one of your essayists at this our ninety-second annual convention; but as you have seen fit to detail me for this task I will not shirk it, as I should be pleased to do, but endeavor to present for your consideration a few ideas concerning the specialty I have adopted, which I hope may not be without interest to you.

Contrary to the prevailing idea, dentistry is not of recent origin. The Egyptians had specialists in every department, and it is claimed that artificial dentures of wood, ivory, and gold have been found in the jaws of mummies. It has been asserted that fillings of gold have been found in their teeth; but recent investigations by several English dentists of high repute tend to throw discredit on this statement, and they incline to the belief that the teeth were only gilded, as oftentimes were their faces, as a means of decoration. On the other hand, there is the authority of Sir Gardiner Wilkinson, who states that he has seen teeth filled with gold in the mouth of a mummy at Thebes, and the fact that Herodotus mentions the existence of both oculists and dentists as specialists among the ancient Egyptians, makes the statement appear probable.

Hippocrates, 500 B. C., and Aristotle, 350 B. C., wrote largely on the teeth,

Celsus, 100 B. C., recommended the use of the file for removing the sharp edge of carious teeth.

Galen, A. D., 100, treated the subject more extensively than any ancient author.

Ætius, an Arabian, A. D., 300, discovered the apicial foramen through which the nerves and vessels enter the dental pulp.

From this time until the latter part of the middle ages, the practice of this specialty, like that of surgery, fell into the hands of mechanics. But about the time of the revival of surgery some advantages toward modern dentistry were made. Among the writers of this period were such men as Fallopius, Eustachius, and Pare, later came John Hunter, and others of his day. Dentistry in those days consisted mainly in the construction of artificial dentures, and was practiced as an art, by jewellers—as a century or two before rude surgery was performed by barbers.

During the eighteenth century the practice of this art began in America, but it was soon discovered that for the intelligent and successful treatment of the teeth some anatomical knowledge of these organs and surrounding tissues was necessary, and the better class of those engaged in this calling began the study of their anatomy, physiology, pathology, etc., and some medical men began to engage in the practice.

Societies were organized, journals and text-books published, and about 1839 the first dental college was established. American dentists soon became acknowledged of superior skill the world over. At this time the almost universal remedy for nearly all diseased conditions of the teeth, except simple caries, was the forceps and the substitution of artificial teeth. Now, exposed and inflamed pulps are saved alive; abscessed teeth, atrophied sockets and necrossed jaws, are successfully treated and saved; extraction, except in extreme cases, is considered malpractice.

Dental deformities are corrected ; fractures of the maxillæ, diseased antra, and all abnormal conditions of the mouth, are considered as coming within the province of a thoroughly educated dental and oral surgeon. Not only this, but by proper prophylactic measures in the young, impairment of these organs, is, to a great extent, arrested.

But while scientific dentistry has rapidly advanced, empiricism has not been idle, and it would be impossible to estimate the amount of injury inflicted by the quacks with which the country abounds.

The Society for the Prevention of Cruelty to Animals protests strongly against vivisections for original investigations; would it not be better employed in waging war against the wholesale extraction and mutilation of the valuable organs under consideration ?

The importance of good teeth to mankind is greater than is appreciated by many. Like the eye, ear, tongue, and other special organs, they are designated for a life service, and their preservation contributes valuable service to the human economy, while their premature loss is of serious damage to the whole body.

In a late report of the Odontological Society, of Great Britian, Edward Canton, F. R. S., gives a large number of cases of habitual constipation, with enormous accumulations of fæces in the descending colon and distressing symptoms of all kinds, which were distinctly traceable to absence of, or a diseased condition of the teeth, to such an extent that mastication was not properly performed, and where treatment was without avail, until the masticatory apparatus was put in proper condition, when the constipation was cured. He says, "It is well known that imperfect mastication of food is a common cause of diarrhea, but few medical practitioners appear to be aware that habitual constipation is not unfrequently due to this cause." . . . . . . . . . . .

"It is, in fact, scarcely possible to exaggerate the importance of proper mastication of the food. It should be reduced by the teeth to a complete pulp, and unless so reduced the digestion is sure to be deranged and general lowering of health will follow. The imperfectly digested mass, which passes through the pylorus, does not take up a proper amount of bile, nature's purgative, and the consequences which I have just been describing follow as a matter of course. I have said that imperfect mastication always causes more or less general impairment of nutrition; this is sometimes very marked; the patient continues for some time thin and weak, and at last falls an easy victim to any illness by which he may be attacked. In women this general low state of nutrition greatly predisposes to barrenness. A young lady was brought to me by her husband; she had been married for some time but had no family. She was thin, "nervous," had no appetite, suffered from indigestion when she did eat, was restless at night, and had bad dreams. I asked her if she masticated her food properly, and she answered, "Oh yes;" but, on looking into her mouth I found that her teeth were very badly decayed. I recommended the supply of molar teeth; they were adopted, the lady got stout aud strong, soon became pregnant, and eventually had several children. So marked was the improvement in her health, and so evident the connection between this and the subsequent pregnancies, that when the husband paid me an occasional visit, and I asked after his wife, he used to answer, "Oh, she is quite well, thank you, Mr. Canton, *she does not want any more teeth!*"

Dr. Oliver Wendell Holmes, in a commencement address delivered before the students of the Dental Department of Harvard University, after speaking in his happy strain of the value of the teeth in relation to the beauty of the human countenance, says: "But we must add to this the consideration, that speech is so largely dependent on the perfection of the teeth, that language, we might say, loses a little with every tooth that falls. What can be more painful to witness than the efforts of a hapless friend to bite his consonants out of the alphabet when he is reduced to the condition of the infant, whose boneless gums are unfit for any task but the caressing pressure of the maternal mouthful!" "And then the humbler, but still necessary function of mastication, how much depends upon the ease and perfection with which this is performed! You can tell the state of a village by going to the mill. If it has enough to grind, and grinds it well and cheaply, you will find good farms and well fed people; so if you see a good square jaw, filled with good sound teeth, and moved by a set of muscles that mean business, and do it, you will find in all probability, that they nourish a second frame in man or woman." The teeth and their surrounding tissues, connected as they are through the fifth pair of nerves with the centers of inervation, and the vasomoter system, do undoubtedly, when in an abnormal condition, exert through the ramifications of these nerves a baneful influence through the body. I am continually impressed with the ignorance of the public in regard to this matter of so much value to their general health, that if they could only be made to understand more fully about it, I think they would be saved a great amount of suffering, to say nothing of saving them from bad dental practice, which instead of encouraging the preservation of these useful organs, hasten their destruction and loss. Even physicians are not as well informed on this subject as they might be, to advantage. As medical colleges are now conducted, medical graduates go forth in as great ignorance of diseases of the teeth as do dental graduates of general disease. Lest you opinionated in my views on the subject, let me quote what I head J. Marion, M. D., say at a meeting of the New York Odontological Soeiety. "As to the effects of diseased teeth upon the general health, I wish medical men generally could be better educated upon that point. We are all familar with the fact that decayed teeth frequently cause neuralgia ; and this is the extent of medical education on this point.

[TO BE CONTINUED.]

